# Role of TNF in the Altered Interaction of Dormant *Mycobacterium tuberculosis* with Host Macrophages

**DOI:** 10.1371/journal.pone.0095220

**Published:** 2014-04-17

**Authors:** Uma S. Gautam, Smriti Mehra, Muhammad H. Ahsan, Xavier Alvarez, Tianhua Niu, Deepak Kaushal

**Affiliations:** 1 Division of Bacteriology and Parasitology, Tulane National Primate Research Center, Covington, Louisiana, United States of America; 2 Department of Microbiology, Tulane National Primate Research Center, Covington, Louisiana, United States of America; 3 Department of Comparative Pathology, Tulane National Primate Research Center, Covington, Louisiana, United States of America; 4 Department of Biostatistics and Bioinformatics, Tulane University School of Public Health and Tropical Medicine; 5 Department of Microbiology and Immunology, Tulane University School of Medicine, New Orleans, Louisiana, United States of America; University of Minnesota, United States of America

## Abstract

*Mycobacterium tuberculosis* (*Mtb*) persists within lung granulomas, despite being subjected to diverse stress conditions, including hypoxia. We hypothesized that the response of host phagocytes to *Mtb* experiencing hypoxia is radically altered and designed *in vitro* experiment to study this phenomenon. Hypoxia-stressed (*Mtb*-H) and aerobically grown *Mtb* (*Mtb*-A) were used to infect Rhesus Macaque Bone Marrow Derived Macrophages (Rh-BMDMs) and the comparative host response to *Mtb* infection studied. Mechanistic insights were gained by employing RNAi. *Mtb*-H accumulated significantly lower bacterial burden during growth in Rh-BMDMs, concomitantly generating a drastically different host transcriptional profile (with only <2% of all genes perturbed by either infection being shared between the two groups). A key component of this signature was significantly higher TNF and apopotosis in *Mtb*-H- compared to *Mtb*-A-infected Rh-BMDMs. Silencing of TNF by RNAi reversed the significant control of *Mtb* replication. These results indicate a potential mechanism for the rapid clearance of hypoxia-conditioned bacilli by phagocytes. In conclusion, hypoxia-conditioned *Mtb* undergo significantly different interactions with host macrophages compared to *Mtb* grown in normoxia. These interactions result in the induction of the TNF signaling pathway, activation of apoptosis, and DNA-damage stress response. Our results show that *Mtb*-H bacilli are particularly susceptible to killing governed by TNF.

## Introduction

Tuberculosis (TB) is responsible for over ∼8.7 million new cases and 1.4 million deaths every year [1]. Upon aerosolization, *Mtb* primarily infects pulmonary alveolar macrophages. This causes a robust response that culminates in the formation of the lung granuloma [2], [3]. Within the granuloma, the rapid replication of *Mtb* is arrested due to the direct action of the activated immune cells, as well as due to nutritional restrictions in a granulomatous environment [2], [3]. Hypoxia has long been recognized as a prevalent stress condition that *Mtb* must contend with inside granulomatous lesions [4].

*Mtb* has developed a specialized transcriptional program regulated by the *dos*R (*dev*R)-encoded protein that enables it to persist in hypoxic conditions [5]. Concomitant with the induction of the DosR regulon, the *Mtb* transcriptional and metabolic profile is assumed to undergo a rapid and significant reprogramming in response to hypoxia and other stress conditions prevalent within human granulomas. Clearly, this would cause major changes in the antigenic profile presented by the pathogen and may influence and modulate host-pathogen interaction. Macrophage-*Mtb* interactions induce the transcriptional machinery resulting in the secretion of several proinflammatory cytokines, chemokine, expression of costimulatory molecules and effector molecules, which provide host defense to *Mtb* [6], [7].

Our current study characterized the impact of hypoxia on the response(s) mounted by host macrophages against *Mtb*. Thus, we tested our hypothesis that tubercle bacilli grown *in-vitro* in anaerobic or hypoxic conditions exhibit markedly different interactions with host macrophages relative to actively growing *Mtb*. Rhesus macaque bone marrow derived macrophages (Rh-BMDMs) were infected with *Mtb* strain H37Rv that was either cultured by aerobic shaking (*Mtb*-A) or subjected to prolonged hypoxia (*Mtb*-H). Data suggest that infection with *Mtb*-H causes extensive transcriptional changes relative to infection with *Mtb*-A to modulate the host immune response. Our results indicate that host response to *Mtb*-H is radically different than *Mtb*-A and *Mtb*-H is rapidly killed within host macrophages due to coordination between TNF and apopotosis. Unsurprisingly, RNAi mediated silencing of TNF abrogated the clearance of *Mtb*-H by Rh-BMDMs.

## Materials and Methods

### Mtb Cultures

*Mtb* H37Rv (a kind gift from Dr. David Sherman, Seattle Biomedical Research Institute, Seattle, WA, USA) cultured in Middlebrooks’s 7H9 media supplemented with ADC to logarithmic phase (A_595_ ∼0.3) with shaking (aerobic), henceforth referred to as *Mtb*-A. To subject *Mtb* to hypoxia, *Mtb*-A (from above) were dispensed into screw-capped air tight 15 ml tubes (head-space 0.6), tightly sealed, wrapped with parafilm and left standing at 37°C for 30 days (*Mtb*-H) with control tubes containing methylene blue (1.5 µg/ml) as an indicator of oxygen depletion in parallel [8]–[10]. In order to obtain bacilli adapted to low oxygen after 30 days hypoxia setup, supernatant was thrown-away and the truly dormant bacilli settled at bottom-only were used for Rh-BMDMs infection. Viable number of *Mtb*-A and *Mtb*-H were enumerated by CFU assay on 7H10 plates prior to infection. To disrupt bacterial clumps, all *Mtb* cultures were repeated passage 10 times (using 27/28 gauge needles) prior to infection. As a control, *Mtb*-H was heat-killed (90°C/45 min) and used to infect Rh-BMDMs.

### Isolation of Rhesus Macaque BMDMs (Rh-BMDMs) and Infection with Mtb

Procedures for the generation and maintenance of Rh-BMDMs as well as their infection with *Mtb* and CFU analysis have been described earlier [12], [13]. Briefly, Rh-BMDMs were cultured in IMDM media (Gibco) supplemented with 10% heat-inactivated FBS (Hyclone) and 1% Penicillin/Streptomycin mix (Pen/Strep, Gibco). This media is subsequently referred to as IMDM complete. The cells were then maintained in Multiwell™ TC Plates (Cat. No. 353046, BD Biosciences) and incubated at 37°C in a humidified 5% CO_2_ incubator. Rh-BMDMs were infected at multiplicity of infection (MOI) of 10∶1 (10 bacteria per 1 cell) in all experiments. After 3-hour cells were washed with PBS and further incubated for 1-hour in IMDM complete containing amikacin (200 µg/ml) (zero hr time-point). The infected cells were lysed (0.1% saponin) for CFU assay or added with 1 mL Trizol for RNA isolation. When macrophages were adherent, both floater as well as adherent macrophages were lysed and plated at 10-fold dilutions on 7H10 agar for CFU counts. The infected Rh-BMDMs in remaining wells were further incubated for 4, 24 and 72 hr.

### Immunofluorescence

For confocal microscopy, Rh-BMDMs were grown and infected in chamber slides (Lab-Tek™ II Chambered cover glass, 2 Well, 4.0 cm2 (Cat#155379, Thermo scientific, Nunc), as described earlier [12]. Adherent cells were washed two-times with warm PBS, fixed with 2% paraformaldehyde (Affymetrix) for 1 hour at RT and were either stored at 4°C or directly used for immunostaining. The use of the anti-*Mtb* antibody (Cat#ab905**,** 1∶200 dilution) for detection of *Mtb* is well established and has been used earlier [12]–[16]. An antibody against Ln5 (Cat No. 18-0165 Zymed/Invitrogen Inc, 1∶50 dilution) was used to stain Rh-BMDMs and anti-TNF (Cat No. 558882, BD Bioscience, 1∶10 dilution) for detection of TNF.

### Host Transcriptomics

DNA Microarray studies were performed as described earlier [12]–[16]. RNA Samples from uninfected Rh-BMDMs exposed to control vehicle IMDM complete media (labeled with Cy3) were compared to Rh-BMDMs infected for 24 hrs with either *Mtb*-A or *Mtb*-H suspended in IMDM (Cy5). Rhesus macaque whole genome 4×44k arrays (Agilent Technologies) were used for profiling. Data was analyzed as described earlier [12], [14], [16]–[19]. Briefly, we used GenePix 4000B to scan chip images, GenePix Pro 6.0 to acquire raw data and Spotfire Decision Site for Microarray Analysis (TIBCO-Spotfire Inc) to perform data analysis using protocols that have been developed by us [20]. Chip normalization was based on S^+^ Array Analyzer locally weighted scatterplot smoothing. Database for Annotation, Visualization and Integrated Discovery (DAVID) was used to analyze gene-ontologies differentially included in the transcriptomics experiments as described earlier [19]. Venn diagrams were prepared using the resources available at http://omics.pnl.gov/software/VennDiagramPlotter.php. Microarray data including raw data and images (accession number GSE47163) of the present study can be accessed from Gene Expression Omnibus (GEO).

### Quantitative RT-PCR

**Figure 1:**
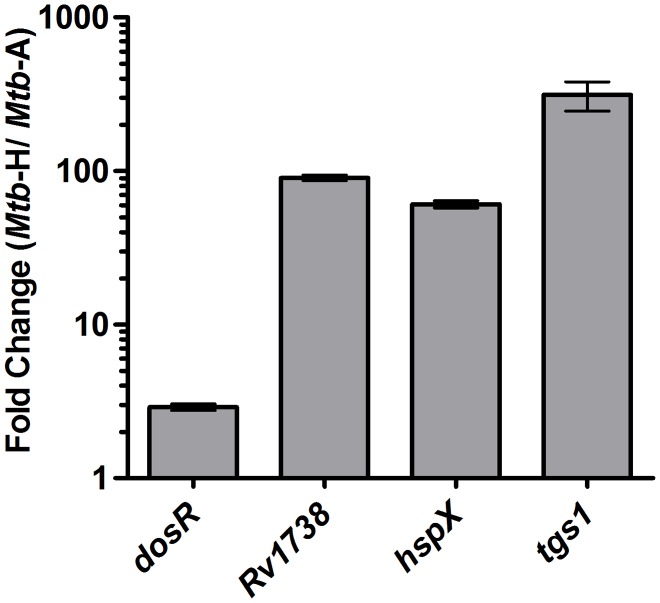
Induction of the DosR-regulon upon culturing *Mtb* in hypoxia. The *devR* transcripts and DevR protein dependent ‘*devR* regulon’ transcripts (e.g. *Rv1738*, *hsp*X and *tgs*1 transcripts, ref. 41) in *Mtb* cultures grown under hypoxic (30 days) versus aerobic conditions is shown. Fold induction of *dos*R *regulon* genes in *Mtb*-H vs. *Mtb*-A bacterial cultures obtained by Real-time RT-qPCR performed in triplicates is expressed as Mean ± standard deviation (SD); for *dos*R 2.90±0.13, *Rv1738* 90.25±3.96, *hsp*X 60.81±3.43 and *tgs*1 are 313.32±67.84. Data were normalized using 16S ribosomal RNA as an invariant transcript and calculated as fold induction using delta-delta Ct method (ΔΔCt) (ref. 21).

Quantitative RT-PCR (RT-qPCR) was used to validate microarray results for a subset of genes. RT-qPCR was performed with cDNA corresponding to 1000 ng DNA-free RNA, using the SYBR green Supermix (Applied Biosystems). A RT-negative (devoid of reverse transcriptase) reaction was used to account for residual DNA if any and relative expression levels were normalized using 18S rRNA as an invariant transcript and data was analyzed using the ΔΔCt method (Figure 1). Additionally, RT-qPCR was also used to identify if *Mtb dosR* regulon genes that were induced during the 30 days hypoxia experiment in *Mtb*-H cultures; 16S rRNA gene was used as an invariant normalization control. Detailed information about oligonucleotides used in RT-qPCR assays is available in Supplement S1.

### Cytokine Assay

Supernatants collected from Rh-BMDMs infected with either *Mtb*-A or *Mtb*-H or IMDM complete medium alone for 24 hours were used for quantification of secreted TNF and IL8 using nonhuman primate (NHP) cytokine-23-milliplex kit (Millipore) according to the manufacturer’s directions.

### Tunel Assay

Tunel assay was performed using *in situ* cell death detection kit, fluorescein (Cat#11684795910, Roche) as previously described [12]. Briefly, The cells fixed in 2% formaldehyde in the chamber slides were washed twice with PBS and added with Tunel reaction mixture (50 µl/well), incubated in humidified atmosphere for 60 min at 37°C. The chamber slides were rinsed three times with PBS before analysis under a fluorescence microscope with an excitation/detection at 450–500/515–565 nm. For quantification, ten fields from each section, each with more than 300 cells were counted under a fixed magnification (corresponding to an area of 0.05 mm^2^) using a TCS-SP2 confocal microscope (Leica Microsystems).

### siRNA Transfection

Transfection was performed as per manufacturer’s instructions (Thermo-Scientific). Briefly, a dose curve 5–25 nM TNF siRNA was combined with appropriate volume of transfection reagent for 20 minutes at RT and added to 12-well plate with 1×10^5^ Rh-BMDMs pre-infected with *Mtb-H*. Finally, all transfections were undertaken in a final volume of 1000 µL, with a final siRNA concentration of 25 nM. The Rh-BMDMs infected with *Mtb*-H were incubated in the absence or presence of small interfering RNA (siRNA) for TNF gene (NM_001047149, Sense: GCGUGGAGCUGACAGAUAAUU, Antisense: UUAUCUGUCAGCUCCACGCUU) or positive control (ON-TARGET*plus* Cyclophilin B Control siRNA, cat #D-001820-10) or negative controls (ON-TARGET*plus* Non-targeting siRNA, Cat #D-001810-10 (pool), for 24 hours. Percentage of Rh-BMDMs undergoing apoptosis after 24 hours of exposure to *Mtb*-H alone (no siRNA) or *Mtb*-H with siRNA quantified by *in-situ* Tunel assay.

### Statistics

Statistical analysis was performed with One-way ANOVA or Student’s t-test with Graphpad prism 6.0b and SAS9.2.

## Results

### Induction of the DosR-mediated Response Program in Mtb Subjected to 30 Days of Hypoxia

The induction of *dos*R and DosR-dependent *Mtb* genes was measured by RT-qPCR on cDNA made from RNA isolated from *Mtb*-A and *Mtb*-H cultures. Induction of *dos*R regulon genes unambiguously shows that true hypoxia was established in *Mtb*-H cultures (Figure 1).

### Effect of Prior Hypoxia on the Ability of Mtb to Infect Primary Macrophages, Intracellular Localization and their Growth

**Figure 2:**
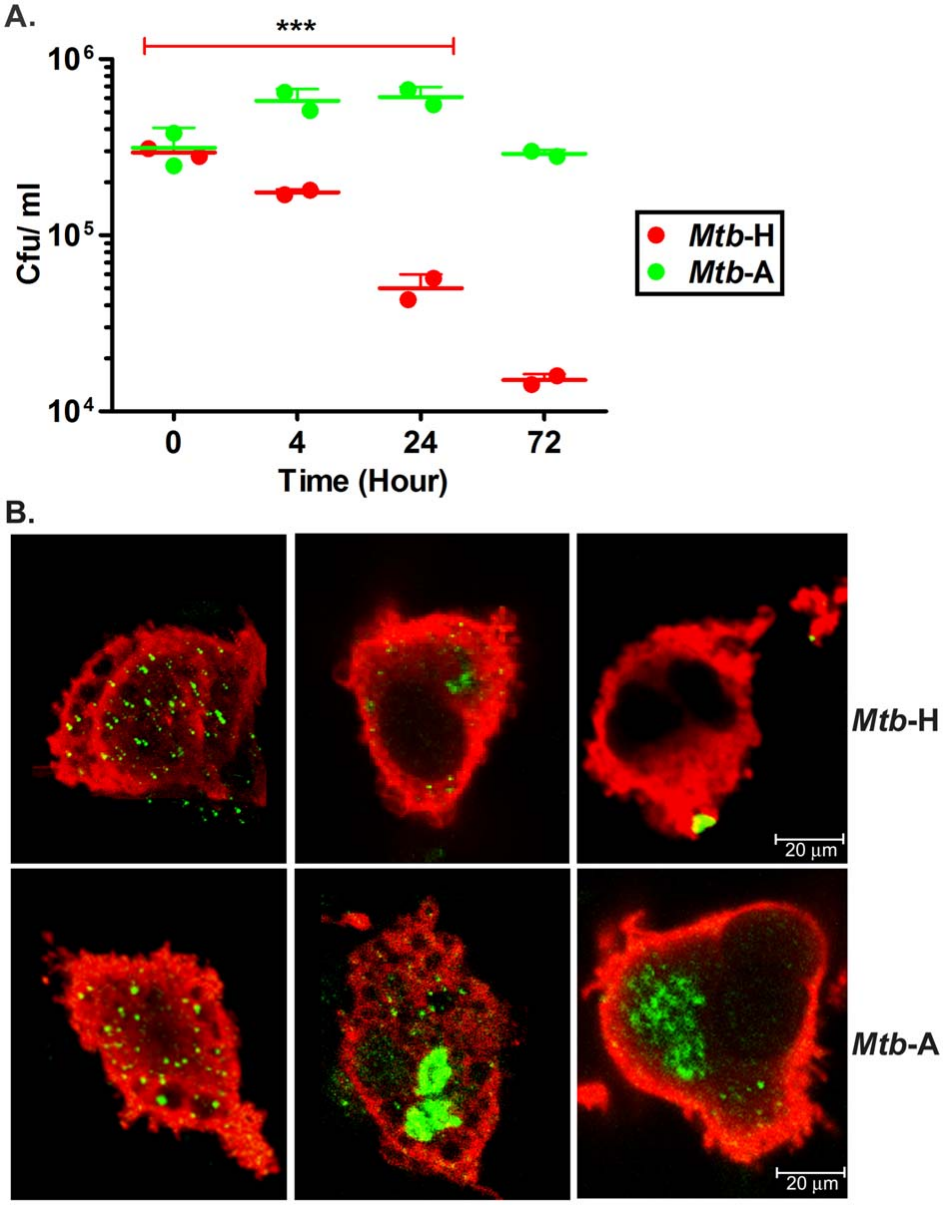
Analysis of the replication of *Mtb*-A and *Mtb*-H in Rh-BMDMs. (**A**) **CFU assay.** Growth progression of *Mtb*-A and reduction of *Mtb*-H during 72 hrs of Rh-BMDM infection measured by CFU analysis is shown. The figure shows the consistent decrease in CFU numbers of *Mtb*-H (***, p<0.005) at 4, 24 and 72 hrs from two biological replicate experiments, with each replicate data comprising of a summarized value from different relevant dilutions and multiple plating. For Statistics, unpaired Student’s t-test (parametric test) was performed using SAS 9.2 (SA Institute, Cary, NC). (**B**) **Confocal Microscopy.** Visual comparison of *Mtb* (green), Rh-BMDMs (red) at 0 hr, 24 hr and 72 hr post infection. For quantitation, bacilli were counted within Rh-BMDMs (more than 250 Rh-BMDMs in all cases) under a fixed magnification using a TCS-SP2 confocal microscope (Leica Microsystems). Total number of bacilli counted in Rh-BMDMs infected with *Mtb*-H, Mean±SD were 750±10 at 0 hr; 136±8 at 24 hr; 34±2 at 72 hr and with *Mtb*-A were 715±20 at 0 hr; 1394±40 at 24 hr; 650±20 at 72 hr. The average relative numbers and SDs determined by CFU assay and Confocal microscopy are shown from at least 3 independent experiments in duplicate or triplicate for each test group.

We have earlier described an *in-vitro* system to study host-pathogen interactions by infecting Rh-BMDMs with *Mtb* [11], [12]. Here we studied if prior exposure of *Mtb* to long-term hypoxia would alter host-pathogen interaction. As readout, we utilized global Rh-BMDM transcriptomics, wherein significant and crucial observations were confirmed at the level of the protein. For Rh-BMDM infection, the number of viable *Mtb*-A and *Mtb*-H was first estimated by CFU assay (allowing us to quantify *Mtb* from three replicates; represented as CFU per mL; *Mtb*-H, 1.07E+07 and *Mtb*-A, 1.15E+07) and were used to infect Rh-BMDMs (10^7^ viable *Mtb* to 10^6^ Rh-BMDMs). The extent of *Mtb* replication was measured by viable cfu count at the end of 4 hr infection (time 0 h) and subsequently 4, 24 and 72 hrs later [11], [12]. Comparable cfu’s were present in the lysates of Rh-BMDMs from two groups at the 0 hr time-point (P = 0.8053) (Figure 2). However, *Mtb*-H was susceptible to killing by Rh-BMDMs and their numbers progressively declined with time. Thus, at the 4 hr time-point, a half-log reduction in bacillary load was observed during infection with *Mtb*-H. By 24 hrs, this difference increased to one-log and by 72 hrs (3 days), the difference in the persistence levels of *Mtb*-A relative to *Mtb*-H was greater than one and a half-log (Figure 2).

Next, we performed multilabel confocal microscopy on Rh-BMDMs infected with *Mtb*-A and *Mtb*-H using an anti-*Mtb* antibody as well as Ln5 to mark *Mtb* and macrophages respectively [11], [12], [16]. Comparable numbers of bacilli could be detected within Rh-BMDMs upon infections with *Mtb*-A or *Mtb*-H at 0 hr time-point (Figure 2). The possibility of a defect in initial uptake of two forms of *Mtb* by Rh-BMDMs was thus ruled out. However, significantly fewer bacilli were observed in Rh-BMDMs infected with *Mtb*-H, relative to those infected with *Mtb*-A at both, 24 hr and 72 hr (3 days) time-points (Figure 2), clearly indicating that prolonged hypoxia diminished the ability of *Mtb* to cope with anti-mycobacterial functions of Rh-BMDMs.

To ensure that the significantly reduced persistence of hypoxia-conditioned *Mtb*-H in Rh-BMDMs was not due to reduced bacterial viability, Rh-BMDMs were infected with heat-killed *Mtb*-H. No bacilli were detected by CFU assay or immunohistochemistry at 0, 4 or 24 hrs post infection of Rh-BMDMs with heat killed *Mtb*-H. The abrogation in the ability of heat-killed *Mtb*-H to infect Rh-BMDMs even at the initial time clearly suggests that hypoxic *Mtb* (*Mtb*-H) was viable at beginning of infection and able to successfully infect Rh-BMDMs. Again, this result clearly shows that significantly reduced persistence of *Mtb*-H in Rh-BMDMs during 3 days of infection is not due to any loss in viability at the beginning of the infection.

### Comparative Global Analysis of the Immune Response of Rh-BMDMs upon Infection with Mtb-A or Mtb-H

**Figure 3:**
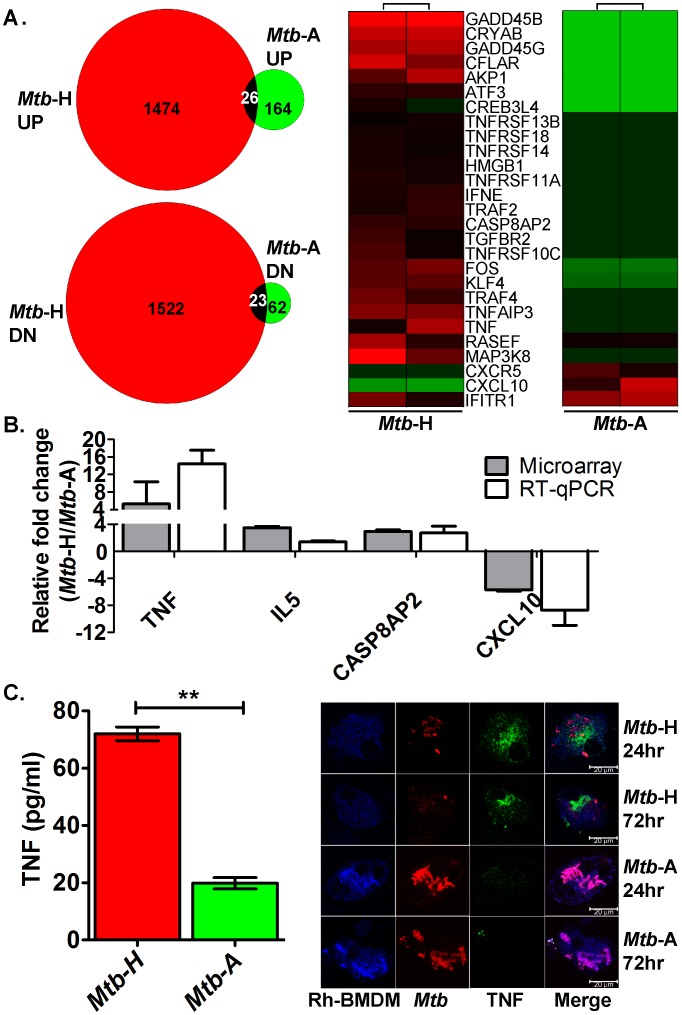
Immune response analysis using microarrays, RT-qPCR, cytokine assay and confocal microscopy. (**A**)**. Microarray**. Venn diagram shows the total number of genes perturbed in *Mtb*-H or *Mtb*-A infected- relative to uninfected-Rh-BMDMs. Total 226 genes (164 genes induced (UP), 62 genes repressed (DN) upon infection with *Mtb*-A; 2996 genes (1474 genes induced (UP), 1522 genes repressed (DN) upon infection with *Mtb*-H). Common genes (from up- or down-regulated gene dataset in both *Mtb*-H and -A group) are shown with overlap. For a description of the common genes e.g. RIPK4 [42], see Supplement S2. Heat-map clusters; green, lower expression; red, higher expression. The data are shown from independent experiments with Rh-BMDMs isolated from two Rhesus macaques. (**B**) **RT-qPCR.** The relative fold change in transcripts **(** *Mtb*-H infected Rh-BMDM to *Mtb*-A infected Rh-BMDM) in microarray (grey bars) and RT-qPCR (white bars) is shown. The relative fold change values (*Mtb*-H to *Mtb*-A) microarray and RT-qPCR (within bracket) are shown below; TNF, 5.3 (14.42); IL5, 3.49 (1.38); CASP8AP2, 2.93 (2.71); CXCL10, −5.7 (−8.7). (**C**) **Cytokine Assay and Multilabel confocal microscopy.** Measurements of TNF in supernatants, *Mtb*-H (red) or *Mtb*-A (green). Experiment was performed in triplicate and values were plotted using GraphPad Prism version 6.0b. The data is statistically significant; Student’s t-test, **P = 0.0027. Confocal microscopy shows secretion of TNF (green signal) detected only in the Rh-BMDMs (blue signal) infected with *Mtb*-H (red signal) (top panels) at 24 hr and 72 hr. The results are shown from Rh-BMDMs derived from two rhesus macaques.

In this study, we utilized global host transcriptomics, wherein significant and crucial observations were confirmed at the level of the protein. Host RNA isolated from uninfected (cells cultured in IMDM complete), *Mtb*-H and *Mtb*-A infected Rh-BMDMs at 24 hr time-point were used to perform rhesus macaque whole-genome transcriptomics as described earlier [11], [12], [15]. The two types of infections produced remarkably different results (Figure 3).

The expression of 226 genes was perturbed in Rh-BMDMs upon infection with *Mtb*-A, comparable to the number of host macrophage genes those were significantly perturbed upon infection with *Mtb* in an earlier study [11]. On the contrary, 2996 genes were perturbed in a statistically significant manner by infection with *Mtb*-H. Thus, *Mtb* subjected to hypoxia elicited an extremely different response from Rh-BMDMs than *Mtb* grown aerobically, both in breadth and in magnitude.

### Specific Differences in Gene-ontologies Perturbed in Rh-BMDMs Infected with Mtb-H Relative to Mtb-A

To better characterize and to understand the biological meaning of the differences between *Mtb*-A and *Mtb*-H infected Rh-BMDMs, we employed Gene Ontology and KEGG pathways biological processes using DAVID (for details of biological processes and their FDR, P values, see Supplement S2) and compared them to results obtained by infection of Rh-BMDMs with *Mtb* earlier [11]. For this purpose, genes whose expression had been modified by at least 2-fold by infection of cells with either *Mtb*-A or *Mtb*-H were included. Briefly, the process “*response to stress*” was significantly included in both datasets. However, infection with the *Mtb*-A and not the *Mtb*-H significantly perturbed the expression of genes involved in defense response and immune response. Processes titled “*response to external stimulus inflammatory response*” and “*chemotaxis*” exhibited a higher level of perturbation in Rh-BMDMs cells infected with *Mtb*-A relative to *Mtb*-H. On the other hand, several biological processes exhibited significantly high levels of perturbation upon infection with *Mtb*-H e.g. “*stress-activated protein kinase signaling*” pathway; NFkβ function e.g. “*regulation of I-kappaB kinase/NF-kappaB cascade*” and “*positive regulation of I-kappaB kinase/NF-kappaB cascade*”; processes involved in programmed cell death e.g. regulation of apoptosis.

Next, we analyzed the differences in gene-expression at the level of individual genes. Infection with *Mtb*-A and *Mtb*-H indeed elicited a differential inflammatory response when specific genes in these categories were analyzed. The expression of several chemokines and their receptors (CXCL10, CXCR5) and cytokine induced genes (GBP1, GBP3, IFI44, IFIH1, IFIT1, IFIT1B, IFIT2, IFIT3) allowed us to identify an IFN gamma-inducible signature or induced to significantly higher magnitude during *Mtb*-A infection (Table 1). This result at the level of the individual genes was in agreement with the results presented earlier at the level of the biological process, where a more significant perturbation of “*defense response*” and “*immune response*” was observed in cells infected with *Mtb*-A (Supplement S2). As shown in Figure 3B, Table 1, Supplement S2, several key immune function genes exhibited differential expression during the two types of infection. Of note, the expression of IL6 was significantly down regulated by infection with *Mtb*-H (Supplement S2). IL6 regulates type 1 interferon pathway genes [21]. Thus, interestingly the expression of IFIT1and IFNE was higher in cells infected with *Mtb*-H, when IL6 levels diminished.

**Table 1:** Functional category of upregulated genes in Rh-BMDMs infected with *Mtb*-H or *Mtb*-A.

Category	*Mtb*-H*	*Mtb*-A*
TNF	TNF (6.5), TNFAIP3 (10.2), TNFRSF14 (2.3), TNFRSF18 (2.8),TNFRSF10C (3.3), TNFRSF11A (2.5), TNFSF13B (2.2),TRAF2 (3.0), TRAF4 (5.5), TRAF6 (2.1), TRAF3IP2 (4.9), LTA (3.0)	
**APOPOTOSIS**	**BID (9.5) CAPN3 (12.4) MAP2K7 (3.1), MAPK8 (3.9), NFKBIA (8.2),** **NRAS (10.2), TNF (6.5**	
INTERFERONS	IFIT1 (4.9)	GBP1 (2.2), GBP3 (9.7), IFI44 (2.7), IFIH1 (3.7), IFIT1 (15.5), IFIT2 (5.2), IFIT3 (4.3), IFIT1B (4.7)
**CHEMOKINES**	**IRF1 (8.9)**	**CXCL10 (9.5), CXCR5 (3.6)**
ACTIVATION OF NEUTROPHILS	F2 (7.8), TNF (6.5), EDN1 (6.3)	HMGB1 (2.4)
**DNA DAMAGE**	**GADD45B (64.2),GADD45G (30.3), DDIT3 (10.95), DDIT4 (4.78)**	**GADD45A (0.433)**
AUTOPHAGY	CFLAR (14.3), ERN1 (12.6), DDIT3 (11.3) BAG3 (7.2), TNF (6.5),CHEK1 (6.4), GAB1 (5.9), DDIT4 (5.0), MAPK8 (3.9), SIRT1 (3.3),TRAF2 (3.0) PPFIA4 (2.8), TICAM1 (2.7), BAIAP2 (2.6), PTGER2 (2.4),SOD1 (2.3), ATG13 (2.2)	
**BINDING OF PHAGOCYTES**	**DLL1 (14.8), F2 (7.8), TNF (6.5), SELPLG (3.4),** **HMGB1 (2.4), TRAF6 (2.1), PTPN6 (2.0)**	
**CHEMOKINE**	**CAMK2D (2.5), FOS (7.2), MAPK8 (3.9), NRAS (10.2)**	
IMMUNERESPONSE	TGFBR2 (3.0), IFNE (2.9), PRDM1 (2.9), PNP (2.9), TXNIP (2.8),TNFRSF18 (2.8), SPIB (2.7) GNA13 (2.7), TICAM1 (2.7),ZFP36 (2.6), DUSP10 (2.6), PTTG1 (2.6), EAF2 (2.6), TNFRSF11A (2.5),ATF2 (2.5), UPP1 (2.5), SCGB1A1 (2.5), HLA-DRB4 (2.5), HMGB1 (2.4),PTGER2 (2.4), EFNB1 (2.4), PRKG1 (2.3), SGK1 (2.3), TNFRSF14 (2.3),EGFR (2.3), IL1RL1 (2.3), NQO2 (2.3), SOD1 (2.3), ZBTB7B (2.3),TNFSF13B (2.2), HBEGF 2.2, TRAF6 (2.1), PTPN6 (2.0)	IFIT1 (15.5),CXCL10 (9.5),IFIT2 (5.2),SPIB (3.9), IFIH1 (3.7), CXCR5 (3.6), POU2F2 (3.4), ISG15 (3.2), RIPK4 (2.9),PLCL2 (2.7), IFI44 (2.7), KIT(2.7) BLNK (2.7), IDO1 (2.7), EDNRB (2.5), TPO (2.4), TPO(2.4), CAMP(2.4),EDN2 (2.3), VCAM1(2.2), IRAK3(2.2)
**INFLAMMATION**	**NLRP3 (21.8), MAP3K8 (20.2), THBD (11.4), TNFAIP3 (10.2), BID (9.5),** **ATF3 (9.4), SPDEF (9.0), NFKBIA (8.2), F2 (7.8), RIPK4 (7.4), NR4A3 (7.1),** **TNF (6.5), DUSP1 (5.6), TRAF4 (5.5), SLC26A1 (5.0), TRAF3IP2 (4.9),** **ID3 (4.4), SLC7A9 (4.3), PTGER4 (4.0), MAPK8 (3.9), IL5 (3.8),** **ZC3H12A (3.7), NFKBIZ (3.7), EGR1 (3.6), TYMP (3.6), PPM1D (3.4),** **SIRT1 (3.3), SERPINC1 (3.2), LTA (3.0), TGFBR2 (3.0), TICAM1 (2.7),** **ZFP36 (2.6), PTTG1 (2.6), EAF2 (2.6), ATF2 (2.5), UPP1 (2.5),** **HMGB1 (2.4), PRKG1 (2.3), SGK1 (2.3), EGFR (2.3), IL1RL1 (2.3),** **NQO2 (2.3), SOD1 (2.3), TNFSF13B (2.2), TRAF6 (2.1)**	**CXCL10 (9.5), ISG15 (3.2),** **BLNK (2.7), IDO1 (2.7), EDNRB (2.5),** **CAMP (2.4),** **EDN2 (2.3), IRAK3 (2.2)**
NFKB SIGNALLING	MAP3K8 (20.2), NRAS (10.2), TNFAIP3 (10.2), NFKBIA (8.2),BMP4 (6.7), TNF (6.5), PIK3C2A (4.0), MAPK8 (3.9), PIK3CB (3.6),MAP2K7 (3.1), LTA (3.0), ZAP70 (3.0), TRAF2 (3.0), TGFBR2 (3.0),TNFRSF11A (2.5), EGFR (2.3), TNFSF13B (2.2), TRAF6 (2.1)	

*Up-regulated genes in Rh-BMDMs infected with *Mtb*-H or *Mtb*-A by microarray. Fold change values are shown in bracket for each gene. To distinguish, alternate categories are shown in bold text.

Genes belonging to the TNF family, which are also considered pro-inflammatory (TNF, TNFAIP3, TNFRSF14, 18, 10C, 11A, TNFSF13B, TRAF2, 4, 6, 3IP2, LTA), were surprisingly induced to higher levels only in Rh-BMDMs infected with *Mtb*-H (Table 1). The up regulation of TNF was noted in Rh-BMDMs infected with *Mtb*-H compare to *Mtb*-A in microarray, RT-qPCR, cytokine assays, confocal microscopy (Figure 3). Higher levels of IL8 (1817 pg/ml) also accumulated in the supernatants of Rh-BMDMs infected with *Mtb*-H compared to *Mtb*-A (127 pg/ml). The expression of several pro-apoptotic genes (BID, CAPN3, MAP2K7, MAPK8, NFKBIA, NRAS and TNF itself) was induced to high-levels only in *Mtb*-H-infected Rh-BMDMs (Table 1). These results indicate that while the NFKB gene ontology is significantly overrepresented (fold change −1.135, P = 0.0055) in the Rh-BMDMs infected with *Mtb*-H, NFKB signaling pathway itself is not activated. This is further supported by the lack of induction of IL6 and IL1β, major NFkβ targets [22], whose expression was significantly repressed during infection with *Mtb*-H rather than *Mtb*-A. Further details about the genes that exhibited perturbed expression are available in Supplement S2.

Yet other important genes from DNA-damage inducible genes category (GADD45B, 64.2-fold; GADD45G, 30.3-fold) were induced in Rh-BMDMs infected with *Mtb*-H but not *Mtb*-A (Figure 3A). These results are strongly corroborated by the observation that Rh-BMDMs infected with *Mtb*-H but not *Mtb*-A undergoes significantly high levels of apoptosis. The extent of apoptosis was examined next by Tunel assay.

### Detection of Apoptosis in Rh-BMDMs Infected with Mtb

**Figure 4:**
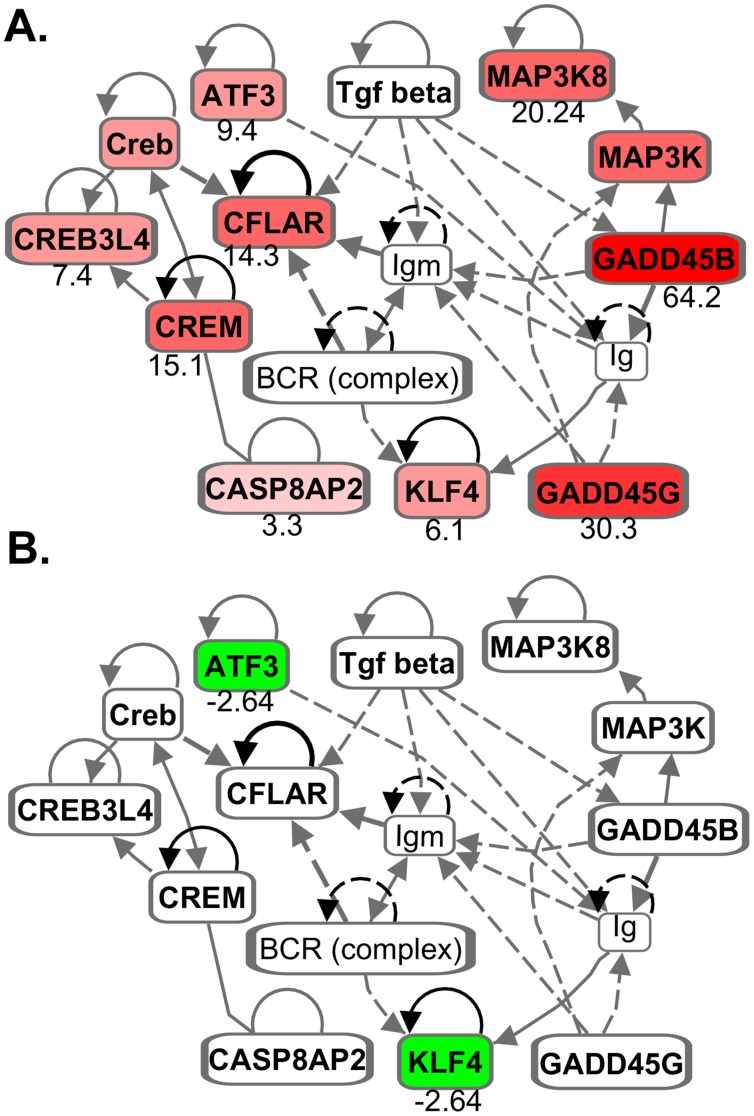
Signaling pathways involving growth arrest and DNA damage inducible genes. Various genes (e.g. GADD45B 64.156 fold; GADD45G, 30.299 fold; MAP3K8 20.242 fold etc.), up-regulated in Rh-BMDMs in response to *Mtb*-H (**A**) are down-regulated in Rh-BMDM infected with *Mtb*-A (**B**). Grey lines represent multiple steps. ATF3-Activating transcription factor 3; CREB-cAMP response element-binding protein; CREM-cAMP response element modulator; KLF4-Kruppel like factor 4 that interact with CREB-binding protein [43]; CFLAR- CASP8AP2 and FADD like receptor. Figures were made using Ingenuity IPA software (Ingenuity Systems, Inc. USA).

**Figure 5:**
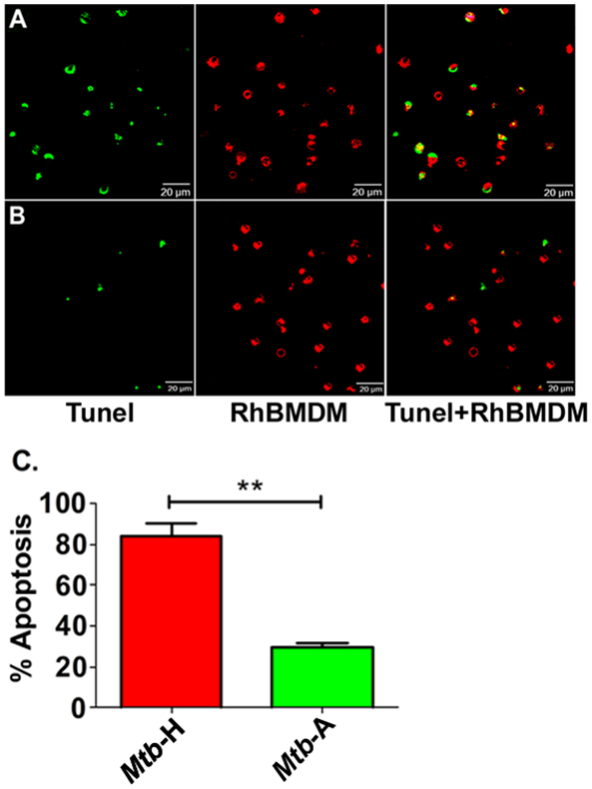
Functional validation of differential apoptosis in Rh-BMDMs infected with *Mtb*-H or *Mtb*-A by Tunel assay. Apoptotic cells show Tunel-positive nuclei (green) and Rh-BMDMs (red). Representative results are shown for Rh-BMDMs infected with *Mtb*-H (**A**) and the *Mtb*-A (**B**) at the 24-hr post-infection. (**C**) Graph (from Figure 1A and B) shows percentage of Rh-BMDMs undergoing apoptosis after 24 hours of exposure to *Mtb*-H or *Mtb*-A quantified by in situ Tunel assay. ** Student’s t test, p<0.005.

Since the gene expression of various components of the apoptotic pathway (Figure 4) was induced to higher levels in Rh-BMDMs infected with *Mtb*-H relative to *Mtb*-A, Confocal microscopy based Tunel assay was performed to examine Rh-BMDM apoptosis (Figure 5), relative to uninfected cells, as a function of time. Significantly more apoptosis, as measured by Tunel positivity, was observed for Rh-BMDM cells infected with *Mtb*-H at 24 hrs (Figure 5A), relative to cells infected with *Mtb*-A (Figure 5B). Quantitative data from counting Tunel positive cells from ten fields in each section exhibited a statistically significant difference (Figure 5C).

### Effect of TNF Silencing on Mtb Growth

**Figure 6:**
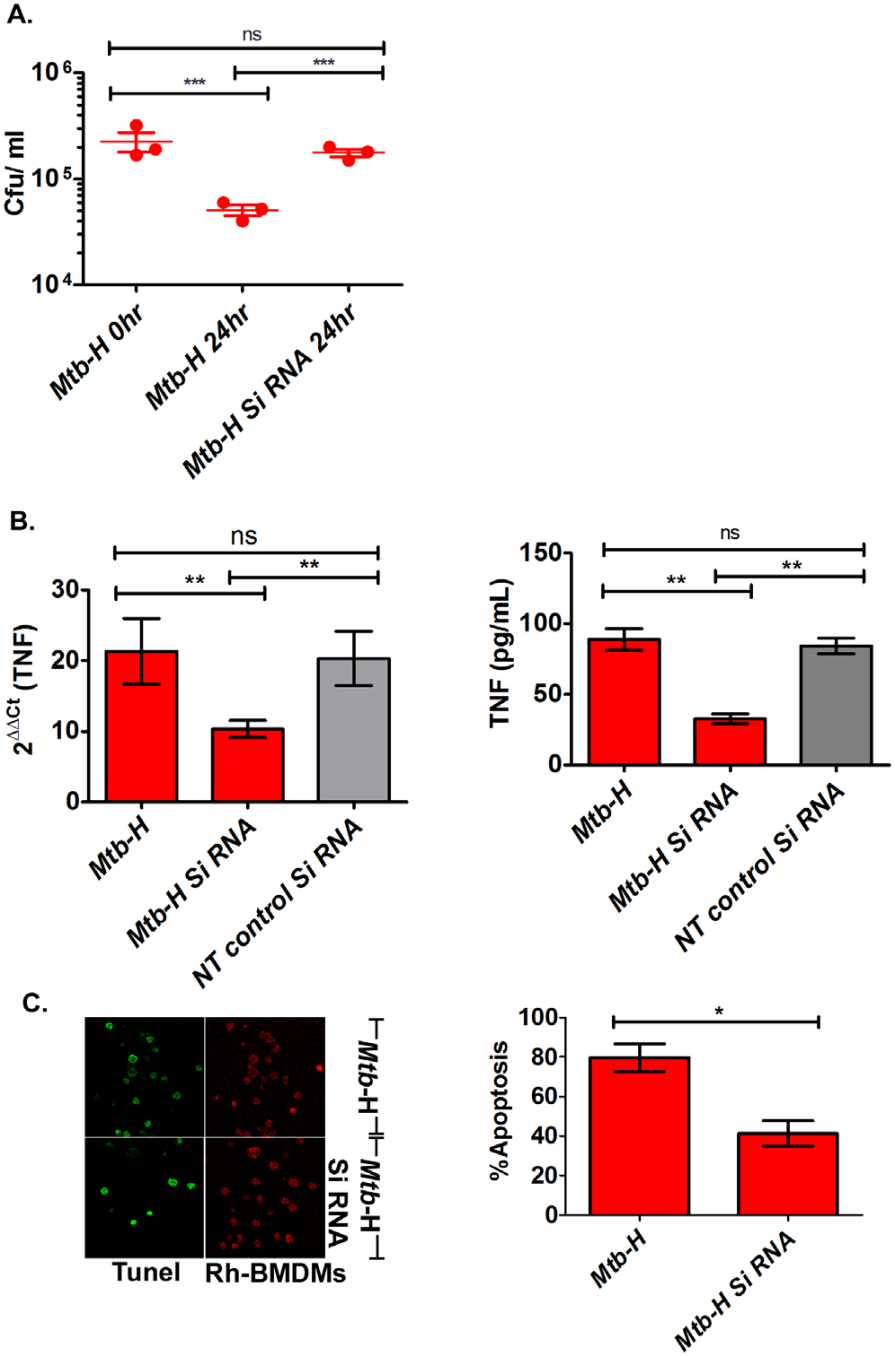
TNF gene silencing. Rh-BMDMs infected with *Mtb*-H in the absence or presence of small interfering RNA (siRNA) for TNF gene is shown by A) CFU assay. B) RT-qPCR, Cytokine assay. C) Tunel assay. Percentages of Rh-BMDMs undergoing apoptosis (left panel) after 24 hours of exposure to *Mtb*-H alone (no Si RNA) or *Mtb*-H with SiRNA quantified (right panel) by in situ Tunel assay are shown from 3 independent experiments. Statistics; one way analysis of variance (Bonferroni’s multiple comparison test), **, P<0.005, ***, P = 0.008; unpaired student’s t-test *, p<0.02.

To pinpoint the effect of TNF on the rapid clearance of *Mtb*-H in Rh-BMDMs, we silenced the expression of TNF by RNAi for 24 hrs and studied its effect on bacterial burden in Rh-BMDMs pre-infected with *Mtb*-H. Specifically, Rh-BMDMs were infected with *Mtb-*H (10 bacteria per 1 cell), and treated with either TNF-specific or negative non-target control siRNA as described earlier [21]. To detect changes in TNF levels in Rh-BMDMs infected with *Mtb*-H, we employed RT-qPCR. PPIB and non-target siRNA were used as the silencing reference standard. The difference between PPIB-transfected Rh-BMDMs and the corresponding negative control was used to calculate the percentage of PPIB mRNA that remained in Rh-BMDMs. The PPIB siRNA knocked down the PPIB mRNA by >80% in Rh-BMDMS infected with *Mtb*-H (data not shown). Similarly, TNF siRNA induced the reduction of TNF mRNA and protein levels, by 52% and 60% respectively in Rh-BMDMs infected with *Mtb*-H (Figure 6B). Importantly, a significant reduction in apoptosis (52%) was also noted in presence of siRNA (Figure 6C, more than 10 fields were counted and were plotted as % apoptotic cells). Interestingly, CASP8AP2 levels were also reduced in RT-qPCR (56% reduction). Silencing was performed in triplicate with rhesus macrophages isolated from three independent animals. Administration of TNF siRNA for 24 hours caused a significant increase in CFU levels (P<0.001, Figure 6A). Thus, reduced TNF expression correlated with increased CFU counts during *Mtb* infection, indicating a role of TNF in the control of *Mtb* replication.

## Discussion

Hypoxia is a critical environmental factor that *Mtb* is faced with during the infection cycle in host cells [23], [24]. The *Mtb* response to hypoxia is primarily driven by the transcription factor DosR [25], [26]. Hypoxia-mediated changes in metabolism are believed to alter *Mtb* replication and force it into a dormant or non-replicative persistent mode of survival [27], [28] within human lungs where the pathogen is contained inside caseous granulomas that gradually become hypoxic [29]. While the effect of hypoxia on *Mtb* is well studied, little is known about how hypoxia-stressed *Mtb* affects host cells in vivo. A recent report suggests that hypoxia plays a role in inducing innate immune responses governed by beta-defensins [30], dormant bacilli can infect human THP1 cells but declined in numbers over 3 days post-infection [31] again confirming our results (present study) and those obtained with human macrophages [32]. However, a global host transcriptional response elicited by the two different physiologically different forms of *Mtb* has not been reported in detail.

In the present study, we compared the transcriptional responses of Rh-BMDMs; *Mtb*-H versus *Mtb*-A. The interaction of Rh-BMDMs with *Mtb* showed that initial uptake of *Mtb*-H or *Mtb*-A by macrophages is not different (Figure 2A). However, the restriction of bacterial growth, the key function that defines the quality of an effective immune response, was significantly altered in Rh-BMDMs infected with *Mtb*-H during 3 days of infection where a significant reduction in bacillary load was noted. These findings provide new insights on the interaction of hypoxia-stressed bacilli with macrophages *in-vivo*. Particularly interesting are our results, which show that *Mtb*-H perturbs the host transcriptome in a significantly different manner. Thus, TNF and apoptosis related genes are highly expressed in Rh-BMDMs infected with *Mtb*-H relative to *Mtb*-A. While other mechanisms that may be involved in the control of *Mtb*-H infection can not be ruled out, it is interesting to note that macrophages infected with avirulent *Mtb* undergo apoptosis at significantly higher levels [33], [34]. Since apoptosis is essential for the *in-vivo* control of *Mtb,* our data suggests that *Mtb*-H may be recognized by host macrophages as less virulent form with induction of TNF program. In addition to further substantiate, the down-regulation of TNF by siRNA was concomitant with an increase in bacterial burden after 24 hours of gene silencing, indicating correlation between TNF and *Mtb* persistence (Figure 6). Our results showed that macrophages regulate TNF production and apoptosis for *Mtb* containment. Similarly, it has been previously shown that macrophages infected with non-pathogenic mycobacteria e.g. *Mycobacterium smegmatis*, substantially enhanced expression of TNF relative to macrophages infected with pathogenic mycobacteria [35]. These authors concluded that pathogenic mycobacteria result in inefficient p38 MAPK activation on account of reduced TNF expression. Similarly, adipocytes [36], human alveolar macrophages [37] infected with avirulent *Mtb* strain H37Ra significantly induced high level of TNF and apoptosis compared to virulent strain H37Rv.

TNF is known to activate JNK pathway and subsequently the apoptosis loop [38]. Of note, GADD45 genes are critical stress sensors of this process and positively mediate apoptosis induction by cytokines [39] via JNK pathway [40]. The expression of these genes was highly up regulated in Rh-BMDMs infected with *Mtb*-H (present study). Our results are consistent with a previous study [33]–[35] and provide the additional point to the fact that *Mtb* subjected to prolonged hypoxia may interact differently with host phagocytes relative to aerobic-*Mtb*. However, the mechanism(s) involved in regulation of these variations in response to the pathogen are not totally understood.

In conclusion our data indicates that hypoxia-conditioned but not aerobically-grown *Mtb* are rapidly killed by activated host macrophages. This is concomitant with a highly altered phagocyte response elicited by *Mtb*-H, characterized primarily by high levels of TNF and apoptosis, a critical determinant for containing *Mtb* infection. Our results suggest a correlation of possible mechanism of *Mtb* containment *in* *vivo* and provide new insights on host-elicited variations in gene ontology to two different physiological forms of *Mtb*. Our current study did not examine the effects of hypoxia on either host macrophage function or their ability to kill *Mtb*. These experiments constitute future studies underway in our laboratory. We also hope to unravel the specific pathways utilized by TNF to preferentially kill *Mtb*-H.

## Supporting Information

Supplement S1 **Primers used in this study.** The nucleotide sequence of forward and reverse primers used in this study is shown in 5′-3′ orientation. ‘*Mtb*’ means, primers derived from *Mycobacterium tuberculosis* genome and ‘Rh’ means, primers derived from Rhesus Macaque genome.
(DOCX)

Supplement S2 **Gene/Gene ontology in Rh-BMDMs infected with** ***Mtb*** **.** Various gene ontology identified in Rh-BMDMs infected with *Mtb*-A (excel sheet 1); *Mtb*-H (excel sheet 2); comparison list of genes (excel sheet 3), common genes, (excel sheet 4) identified in Rh-BMDMs, *Mtb*-H vs. *Mtb*-A.
(XLS)
