# Supplementary material for: Role of TNF in the Altered Interaction of Dormant Mycobacterium tuberculosis with Host Macrophages
Source: PLoS One. 2014 Apr 17;9(4):e95220. doi: 10.1371/journal.pone.0095220 (PMC3990579; doi:10.1371/journal.pone.0095220)
Supplement: Supplement S1 — Primers used in this study. The nucleotide sequence of forward and reverse primers used in this study is shown in 5′-3′ orientation. ‘Mtb’ means, primers derived from Mycobacterium tuberculosis genome and ‘Rh’ means, primers derived from Rhesus Macaque genome. (DOCX) [file pone.0095220.s001.docx]

| **Primer** | **Primer sequence (5’-3’)** | **Application** |
| --- | --- | --- |
| *Mtb dos*R-F | TATCAGGCCTTACCGACCAG | RT-qPCR |
| *Mtb dos*R-R | AACCGCGACACGTAGTTCTT | RT-qPCR |
| *Mtb Rv1738*-F | CGACGAACACGAAGGATTGAC | RT-qPCR |
| *Mtb Rv1738*-R | GATCTCGGGGACGTTGCGGT | RT-qPCR |
| *Mtb hsp*X-F | CGACAAGGACGTCGACATTA | RT-qPCR |
| *Mtb hsp*X-R | CCTTGTCGTAGGTGGCCTTA | RT-qPCR |
| *Mtb tgs*1-F | GGAAATTCGACGTCACCATC | RT-qPCR |
| *Mtb tgs*1-R | AACACGGTTATCGGTCTTGC | RT-qPCR |
| *Mtb* 16S-F | CCTGGGAAACTGGGTCTAATAC | RT-qPCR |
| *Mtb* 16S-R | CTCATCCCACACCGCTAAAG | RT-qPCR |
| Rh_RT TNF 5’ For | GTGGCAGGCGCCACCACGCTC | RT-qPCR |
| Rh_RT TNF Rev | GATCTGACTGCCTGAGCCAGAGG | RT-qPCR |
| Rh_RT IL5 For | CTGTGCAAGGGGGTACTGTGGAAA | RT-qPCR |
| Rh_RT IL5 Rev | GGTTTACTCTCCGCCTTTCTTCTCC | RT-qPCR |
| Rh_RT CASP8AP2 For | TCAAATGAGGATAGTCGAAGAGGAAG | RT-qPCR |
| Rh_RT CASP8AP2 Rev | GTTCACCATCACCACAGCCAGGAC | RT-qPCR |
| Rh_RT CXCL10 For | GCCATTCTGATTTGCTGCCTTGTC | RT-qPCR |
| Rh_RT CXCL10 Rev | CAGGTTGATTACTAATGATGCAG | RT-qPCR |
| Rh_PPIB For | GGAGAGCACCAAGACAGACAGC | RT-qPCR |
| Rh_PPIB Rev | GGTCCTACTCCTTGGCGATGGC | RT-qPCR |

**Supplementary Table S1. Primer used in this study**

*Mtb*: *Mycobacterium tuberculosis* genes specific primers

Rh: Rhesus Macaque genes specific primers
